# Targeted individual exercise programmes for older medical patients are feasible, and may change hospital and patient outcomes: a service improvement project

**DOI:** 10.1186/1472-6963-8-250

**Published:** 2008-12-10

**Authors:** Jo Nolan, Susie Thomas

**Affiliations:** 1 Physiotherapy Department, Flinders Medical Centre, Adelaide, South Australia, Australia; 2 Rehabilitation and Ageing Studies Unit, Flinders University, Adelaide, South Australia, Australia

## Abstract

**Background:**

The aim of this project was primarily to assess the feasibility of individual exercise programs for older hospitalised patients at risk of functional decline, and secondarily to evaluate impact on discharge outcomes.

**Methods:**

Design: Cohort service improvement project

Setting: 500 bed acute metropolitan hospital

Subjects: Patients aged 70 and older admitted to Flinders Medical Centre under the general medical, aged care and respiratory units from June to November 2006, at intermediate or high risk of functional decline, and able to commence exercise within 48 hours of admission

Intervention: Functional Maintenance Program (FMP); an individually tailored exercise program to maintain functional mobility, prescribed and progressed by a physiotherapist, and supervised by an Allied Health Assistant (AHA), provided in addition to usual physiotherapy care

Outcome measures: Feasibility (number of admissions suitable, commencing and complying with FMP). Impact (length of hospital stay (LOS), Aged Care Assessment Team (ACAT) referrals and approvals, hospital readmissions within 28 days, and functional mobility (Elderly Mobility Scale))

Data Analysis: Descriptive and logistic regression analysis

**Results:**

Of 1021 admissions of patients aged 70 or older to general medical, aged care and respiratory units, 22% (n = 220) were identified within 48 hours as suitable for FMP: 196 (89%) commenced FMP within 48 hours of admission (FMP patients); 24 (11%) received usual physiotherapy (usual care patients). Feasibility of individually tailored exercise programs for older medical patients was supported by high uptake (89%), low withdrawal (17%) shown by those who commenced FMP, and good compliance with exercise sessions (70%). Logistic regression analysis showed a statistically significant decreased likelihood of referral for nursing home admission (OR = 0.228, 95% CI 0.088–0.587) and decreased likelihood of approval for admission to residential care (OR = 0.307, 95% CI 0.115–0.822) in favour of FMP. Although trends of an average 15.7% LOS reduction, 8% fewer readmissions and improved functional mobility were demonstrated in favour of FMP patients, these results were not statistically significant.

**Conclusion:**

It is feasible to identify older medical patients likely to benefit from an exercise program to maintain functional abilities, and to commence within 48 hours of admission.

## Background

Over the next 50 years it is predicted that as the proportion of the population over 65 continues to rise [1], the demand for hospital bed days and pressure on infrastructure and staffing will also expand [2]. Currently, half of all acute hospital beds are occupied by people over 65 years of age [3], and it is projected that the demand for hospital bed days will grow faster than population growth, with the proportion of bed days devoted to older people increasing to over 70% by 2050 [2].

Older medical patients are at increased risk of deconditioning and functional decline during hospital admission [4]. They are also more likely to have an increased length of stay in hospital, more readmissions, and more iatrogenic complications, when compared with younger age groups [5]. A loss of functional independence has the potential to lead to an increased burden of care, a need for community services and/or residential care [6].

In Australia, exercise has been advocated for hospitalised older patients to prevent complications of hospitalisation including a decline in functional mobility [7]. In previously reported trials, exercise has been provided to all participants [8], rather than being targeted to those most likely to benefit. In a previous pilot randomised controlled trial, patients considered at risk of functional decline (based on the clinical discretion of the treating physiotherapist) who received individually prescribed exercise programs, showed a trend towards decreased length of stay (on average 2.91 days shorter from enrolment in the study) although there was a prolonged delay in commencing exercises [9] possibly suggesting that the intervention was rehabilitative rather than preventive in nature.

To overcome issues previously identified in studies of exercise intervention in older medical patients, this service improvement project aimed to commence the intervention with patients likely to remain in hospital long enough to participate and benefit, before functional abilities were compromised due to hospitalisation, and to target those with an objectively identifiable risk of functional decline. It was important to determine whether this was feasible in the acute hospital setting. Operationally it was decided that the intervention should be provided to those at higher risk of functional decline, who could commence within 48 hours of admission and were likely to remain in hospital for at least 72 hours, and where Allied Health Assistant (AHA) staff were available to deliver the program. For the purpose of this project greatest risk of functional decline was objectively identified using the Hospital Admission Risk Profile (HARP) which scores risk of functional decline based on age, cognitive function and dependence in activities of daily living in the two weeks prior to admission [6].

Therefore the aims of the project were to:

1. Determine if it was feasible to identify patients at risk of functional decline and commence an exercise intervention to prevent functional decline (functional maintenance program (FMP)) within 48 hours of admission

2. Determine the impact of FMP on length of stay and discharge outcomes (referrals and approvals for residential care, 28 day hospital readmission rates) and functional mobility for identified patients.

## Methods

### Design overview

This project was conducted over 20 weeks from June to November 2006, with 220 older medical patients at higher risk of functional decline during admission to a 500 bed acute teaching hospital. These patients received FMP based solely on availability of Allied Health Assistant (AHA) staff to commence FMP within 48 hours of admission. Those who were otherwise equally suitable but who were unable to commence within 48 hours solely due to limited staff resource received usual physiotherapy care, and were considered the usual care group for analysis of the impact of FMP. The Flinders Clinical Research Ethics Committee reviewed and approved the project protocol – Research Application 229/08.

### Patient identification and suitability for FMP

Based on daily computer generated printouts of admissions aged 70 and older to general medical, aged care and respiratory units, the case notes of all patients were screened by the project physiotherapist to determine suitability for FMP. Patients needed to have been referred to physiotherapy, be able to carry out a supervised exercise program targeted at maintaining functional mobility and to reside either in the community or low level residential care. Therefore those who were not normally ambulating, had severe cognitive impairment, a primary diagnosis limiting exercise (CVA or fracture), were non-English speaking, palliative or residing in high level care prior to admission were ineligible. Due to limited staffing and funding, baseline mobility outcome measures and prescription of the exercise intervention were the responsibility of staff physiotherapists, who provided usual physiotherapy for all patients. To target those patients at greater risk of functional decline and therefore more likely to benefit from a functional maintenance program, the HARP tool was utilised to identify those at intermediate or high risk of functional decline [6]. Specifically this included patients aged 65 years or older who required assistance to perform two or more ADL's, patients 75 years or older with impaired cognition (MMSE < 15), and all patients aged 85 years or older. Patients were withdrawn from FMP if their medical condition changed in a way that made the intervention of additional exercise inappropriate i.e. new diagnosis of palliation, CVA or admission to ICCU.

### Measurements and procedures

Feasibility of the project was operationally defined by:

1. The number of patient admissions able to be screened for participation in the program

2. The number of potentially suitable patient admissions identified during the project period, able to participate in the intervention within 48 hours of admission and likely to be hospitalised for at least 72 hours

3. The implementation rate of FMP for participating patients

4. The compliance rate of patients with FMP

5. The number of EMS outcome measures able to be collected.

Following initial eligibility screening by the project physiotherapist, ward physiotherapists were informed of those who were suitable for FMP.

Functional mobility was measured using the Elderly Mobility Scale (EMS) [10] and scored by ward physiotherapists on admission and discharge. This may have involved different physiotherapists. Patient participation in the program was recorded by AHA on a daily basis.

The project physiotherapist used the Inpatient Separation Information System (ISIS) to obtain hospital data including length of stay (LOS), 28 day readmission rates and Aged Care Assessment Team (ACAT) referrals and approvals (required for admission to residential care i.e. nursing home and some transitional care services (care services provided in the community to assist older people). Discharge destination was obtained from ward physiotherapists and confirmed against ISIS data, and case notes were used to determine any change in usual accommodation.

### Allocation to participate in FMP

For the purpose of the project, suitable patients participated in FMP and were considered part of the FMP group when AHA staff were available and had capacity to start FMP within 48 hours of admission. Those patients who were equally suitable but unable to commence within 48 hours of admission received usual physiotherapy care and were considered part of the usual care group. Therefore, the control group consisted of patients with the same profile, who were unable to access the intervention. All patients received usual medical and physiotherapy care during their admission.

### Functional Maintenance Exercise Program

Prior to commencement of the project, a group of experienced, senior physiotherapists selected 118 exercises from the VHI program, specifically targeting strength, balance and mobility which were appropriate for a range of acutely hospitalised older patients (see Additional file 1). This selection of exercises was used for prescription of FMP exercises, using equipment readily available in hospital wards eg bed or chairs, and with the option of using weights for resistance where appropriate as has previously been used at this hospital [9]. Based on the findings of the initial physiotherapy assessment of each patient, the usual physiotherapist prescribed FMP exercises in addition to usual care, aimed at maintaining and improving the functional mobility of the individual patient. Factors including individual patient's mobility status, strength, endurance, cognitive ability, medical condition and fatigue were taken into consideration for exercise prescription. In this population, the programs were limited to maximum 30 minutes duration and on average, 6 to 8 exercises with approximately 8 to 12 repetitions were prescribed. If it was appropriate for an AHA to mobilise the participant on their own, then functional walking (exercise number 104), was included. The FMP was supervised through every repetition of every exercise for each patient, once a day, over a six day period (Monday to Saturday) by AHAs, employed 1.5 FTE, and the exercises were progressed or modified as appropriate under the guidance of the treating physiotherapist.

### Statistical Analysis

Data was entered into SPSS 14.0 and the program was used to calculate for comparison between the usual care and FMP groups. Models for analysis by logistic regression were built based on a priori hypotheses regarding variables of interest including age, gender and patient clinical complexity level (PCCL) which is a measure of the cumulative effect of a patient's complications and comorbidities, and is calculated for each episode from the clinical complexity level values assigned. PCCL scores range from 0 (no clinical complexity effect) to 4 (catastrophic clinical complexity effect [11]).

## Results

### Feasibility

During the 20 week project period, 1021 patients were admitted to FMC aged 70 and older under the general medical, aged care and respiratory units, of whom four (0.004%) could not be screened as case notes were not available.

The screening process identified 78% (n = 801) of patient admissions to general medicine, aged care and respiratory units aged 70 years and older were not suitable for FMP, most commonly because they were discharged prior to screening (16%), were admitted from high level care (11%) or were not referred to physiotherapy (10%). Further details are shown in Table 1.

**Table 1:** Reasons for ineligibility for Functional Maintenance Program

**Reason for ineligibility**	**%**	**Number (n =)**
Non-ambulatory	0%	3

Notes not available	0%	4

Requiring psychiatric intervention	1%	8

Deceased	1%	11

Non English Speaking	2%	17

Palliative	2%	19

Dementia	2%	20

Transferred to ICCU	2%	22

Transferred to another hospital	2%	24

Unco-operative	3%	29

Medically unstable	4%	42

Diagnosis contraindicates FMP	4%	43

Transferred under another unit	5%	51

Documented to be D/C within 24 hrs	5%	55

Low risk of functional decline	8%	78

Not referred to physiotherapy	10%	101

High Level Care	11%	109

Discharged prior to screening	16%	165

**TOTAL**	**78%**	**801**

**Figure 1:**
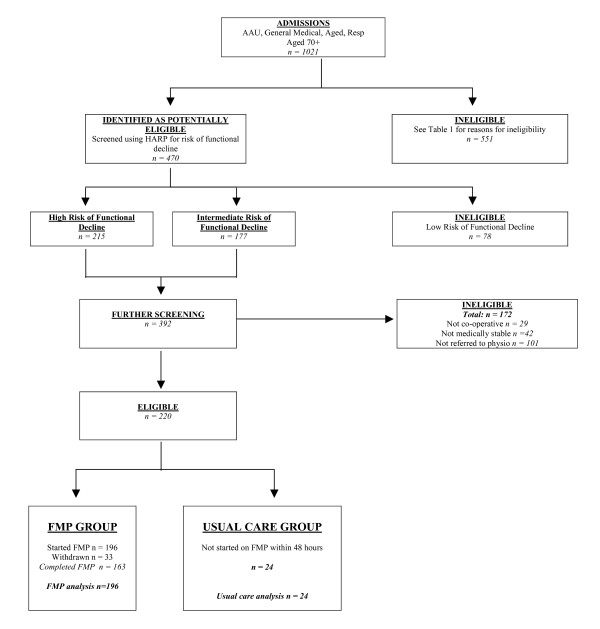
Project profile: flowchart showing the movement of patients through each stage of the project.

This screening process eliminated 32% of all patient admissions (n = 331) in the first instance, with further screening for risk of functional decline using HARP being undertaken which excluded those at low risk (7.6% of all admissions, n = 78). The final phase of screening of those at intermediate or high risk of functional decline eliminated further unsuitable patients e.g. medically unstable or not referred to physiotherapy. Figure 1 shows the project profile and table 2 describes group characteristics.

**Table 2:** Group characteristics

	Age (years)	Gender female:male	Baseline EMS	Discharge EMS	PCCL
"FMP Group" n = 196	83.16 +/- 8.4	133f:63m (68% f:32% m)	8.42 (n = 163)	14.37 (n = 163)	2.49 +/- 1.5

"Usual Care" n = 24	85.42 +/- 5.69	16f:8m (67% f: 33% m)	7.09 (n = 23)	11.91 (n = 23)	2.71 +/- 1.3

### FMP implementation for patient admissions

Two hundred and twenty (22%) of all patient admissions met all suitability criteria and were considered appropriate for consideration of FMP feasibility and impact.

FMP was commenced with 89% of suitable patients (196 of 220) within 48 hours of admission, and those who were otherwise equally suitable but who were unable to commence solely due to resource limitations received usual physiotherapy, and were considered the usual care group (11%, n = 24 of 220). Of the 196 patients who commenced FMP, the majority (83%, n = 163) completed the program, and 17% (n = 33) ceased participation for reasons not associated with the project intervention; 10 patients were discharged before intervention began, seven transferred to ICCU, four were considered to require palliative care, four became medically unstable, four became uncooperative, three died, and one suffered a CVA.

### Compliance with additional exercise program (FMP)

On-going FMP patients (n = 163) received FMP exercises on average for 70% of possible occasions (6.25 of 8.87) during their average LOS of 10.01 days. The most common reason for not receiving FMP was because the service was not offered on a Sunday (14% of missed occasions on average per admission, an average of 1.2 sessions missed per admission for this reason). Other reasons included the patient not being available (e.g. absent from ward for medical investigations), public holiday, patient medically unwell, patient refusal, or limited AHA staff to deliver the program. There were no reports of missed sessions due to complications of FMP participation eg muscle soreness or tiredness.

### Aged Care Assessment Team (ACAT) referrals and approvals, and readmissions within 28 days

Examined by intention to treat, logistic regression analysis with adjustment for age, sex and complexity showed a statistically significant 78% reduced likelihood of referral for ACAT assessment and a statistically significant 69% reduced likelihood of ACAT approval in favour of the FMP group. There were 8% fewer readmissions within 28 days in the FMP compared to usual care patients, which was not statistically significant with 59% reduced likelihood of readmissions within 28 days in the group who received the FMP, as shown in Table 3.

**Table 3:** Analysis for Aged Care Assessment Team (ACAT) referrals and approvals and readmission within 28 days

	**Usual Care group n (%)**	**FMP group n (%)**	**Odds ratio**	**Z score**	**p-value**	**95% CI**	**Statistical significance**	**Trend in favour of intervention**
**ACAT referrals**	10 (42%)	27 (17%)	0.228	-3.06	p = 0.002	0.089, 0.588	✔	

**ACAT approvals**	8 (33%)	25 (15%)	0.307	-2.35	p = 0.019	0.115, 0.822	✔	

**Readmissions within 28 days**	4 (17%)	14 (9%)	0.412	-1.43	p = 0.153	0.122, 1.389		✔

### Discharge Destination

**Figure 2:**
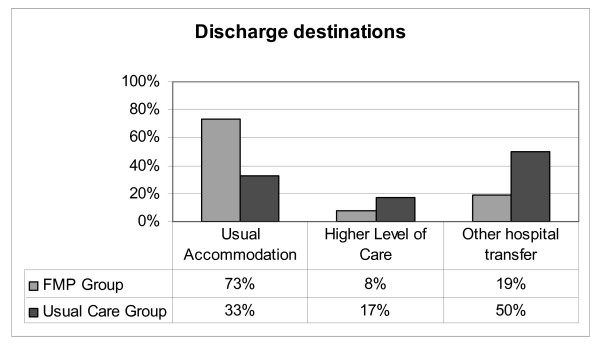
Comparison of discharge destination between groups.

Analysed by treatment received, 73% of the FMP patients (n = 119) were discharged to their usual accommodation compared to 33% of patients who received usual care (n = 8). Eight percent of the FMP patients required a higher level of care on discharge (n = 13) compared to 17% of usual care patients (n = 4). Analysis by logistic regression based on age, gender and patient complexity did not demonstrate statistical significance using either treatment received or intention to treat, as shown in Figure 2.

### Length of Stay

On average, LOS for FMP patients was 1.93 days shorter for the 163 patients who received FMP compared to the 24 patients who received usual care (FMP average LOS = 10.01 +/- 7.88 days compared to the usual care group LOS 11.94 +/- 8.36 days). This represents a 15.7% reduction in average LOS, however logistic regression analysis adjusted for sex and age showed that this mean difference was not statistically significant (OR = 0.412, 95% CI 0.122 – 1.389).

### Elderly Mobility Scale

In this project, both admission and discharge EMS scores were obtained from 84.5% of study participants (186 of 220). Where data were available, scores showed improvement in both groups during hospitalisation, with greater average EMS score improvement for intervention (5.95) than usual care participants (4.82) which was not shown to be statistically significant. Floor or ceiling effects were evident in 7% of EMS scores.

### Power calculation for future research

Based on LOS data from this project, the FMP patients (n = 163) with a 15.7% shorter LOS than those receiving usual care (n = 24) (on average 10.01 +/- 7.88 versus 11.94 +/- 8.36 days), which indicates that for a two-sample comparison of means, 80% power and a common SD of 0.8, the estimated sample size required would be 393 in each group.

## Discussion

This service improvement project has shown that the implementation of an individually prescribed functional maintenance exercise program, targeted to older patients at higher risk of functional decline, is feasible in an acute hospital setting. Whilst a large proportion of patient admissions (78%) were not suitable for the program, they were able to be excluded from consideration in the initial 48 hours of their admission, and the remaining 22% of older medical patient admissions likely to remain in hospital and benefit from a program to prevent functional decline during hospitalisation were identified.

The feasibility of the process in this setting is further supported by the high proportion of suitable patients who were able to commence FMP (89%). The limitation to participation for the remaining 11% was lack of staff resource rather than lack of potential to benefit. In this acute hospital setting the withdrawal rate from FMP of 17% is relatively low, and compliance during hospitalisation of 70% is high, further supporting the feasibility of FMP for older medical, aged care and respiratory patients.

The presence of floor and ceiling effects in 7% of EMS scores is of concern. There was also a limitation of relying on staff physiotherapists to collect outcomes as opposed to a dedicated blinded assessor. For future studies, other mobility outcome measures and blinded assessors should be considered.

The statistically significant reduced likelihoods of referral to, and approval for, residential care admission are also encouraging, as is the 15.7% reduction in length of hospital stay, which might be considered clinically relevant given the pressure on hospital beds and capacity, although it was not statistically significant. However, it must be clearly recognised that the ability to draw strong conclusions from these comparisons is limited by the possibility of selection bias and disparate group sizes.

This intervention has two key differences to exercise interventions reported in other studies [8]. Firstly, this project was specifically targeted to those patients objectively identified as being at higher risk of functional decline, using HARP [6]. It is possible that the impact of the FMP is far greater on those patients at higher risk of functional decline on admission, consistent with increasing age and poor functional ability [12], rather than the intervention being provided to those who are lower risk of functional decline.

Secondly, the intervention was unique; it was specifically prescribed for each patient with a focus on maintenance of functional mobility, provided in addition to usual physiotherapy care, and closely supervised on a daily basis by a trained AHA. Other programs have utilised a generic program [13] or selected from set levels of exercises considered appropriate to patients [8,12].

This project highlights the need for further research into this area, with potential benefits for patients, hospitals, and reduced demand for residential care services. A randomised controlled trial focusing on individually prescribed intervention targeting the most at risk groups of patients and delivered by trained AHA appears warranted. In addition, the impact on other 'at risk' patient populations needs to be investigated, including patients who spend a period of their admission in ICCU, post-surgical, younger respiratory patients, aged orthopaedic patients and those unable to commence within 48 hours of admission. An appropriate assessment tool sensitive enough to detect functional mobility improvements over hospital admissions, which are sometimes short duration, also needs to be identified and utilised.

## Conclusion

• It is feasible to identify older medical patients at risk of functional decline and commence individual exercise programs within 48 hours of admission to an acute hospital

• Feasibility of individually tailored exercise programs for older medical patients is supported by high uptake (89%), low withdrawal (17%) and good compliance with exercise sessions (70%) in the acute hospital setting.

• Consideration of other functional mobility outcome measures which may be more responsive to change is warranted

• Despite the recognised design limitations of this service improvement project which limit the robustness of the project to draw strong conclusions about the impact of additional exercise programs, there does appear to be sufficient benefit to warrant further rigorous evaluation to quantify the impact of an individually prescribed exercise program targeted to acutely hospitalised older medical patients at risk of functional decline during admission.

## Competing interests

The authors declare that they have no competing interests.

## Authors' contributions

All authors were substantially involved in the conception and design of this project. JN and ST drafted the manuscript, contributed to its critical review and approved the final draft.

## Pre-publication history

The pre-publication history for this paper can be accessed here:



## Supplementary Material

Additional file 1 **List of all exercises that could be selected for the Functional Maintenance Program**. The data provided represent all of the exercises that were available for the therapists to choose from when prescribing the Functional Maintenance Program Click here for file
