# Supplementary material for: Targeted individual exercise programmes for older medical patients are feasible, and may change hospital and patient outcomes: a service improvement project
Source: BMC Health Serv Res. 2008 Dec 10;8:250. doi: 10.1186/1472-6963-8-250 (PMC2614989; doi:10.1186/1472-6963-8-250)
Supplement: Additional file 1 — List of all exercises that could be selected for the Functional Maintenance Program. The data provided represent all of the exercises that were available for the therapists to choose from when prescribing the Functional Maintenance Program [file 1472-6963-8-250-S1.doc]

| **Bed based strengthening** | | |
| --- | --- | --- |
| 1. Bench Press^*^ 2. Elbow Extension (supine) ^*^ 3. Scapular Protraction (supine) ^*^ 4. Pelvic Tilt (supine) 5. Ab Crunch | 1. Hip Ab/Adduction (supine) ^*^ 2. Hip Ab/Adduction (side lying) ^*^ 3. Inner Range Quads^*^ 4. Straight Leg Raise^*^ 5. Bridging | 1. Bridging (single leg) ^*^ 2. Rolling 3. Side Lying to Sitting |
| **Bed based flexibility** | | |
| 1. Scapular Retraction 2. Ankle Pump | 1. Ankle Inversion/Eversion 2. Supine Stretch | 1. Knee Roll 2. Knee to Chest (Flexion) |
| **Seated strengthening** | | |
| 1. Elbow Flexion^*^ 2. Shoulder Abduction^*^ 3. Overhead Press^*^ 4. Chest Pull with Resistive Band | 1. Sitting Scoot 2. arm Chair Push 3. Sit to Half Stand 4. Knee Extension^*^ | 1. Knee Extension with Resistive Band 2. Sitting to Standing |
| **Seated flexibility** | | |
| 1. Chin Tuck 2. Neck Flexion 3. Neck Rotation 4. Neck Lateral Flexion 5. Shoulder Circle 6. Shoulder Retraction | 1. Shoulder Shrug 2. Side Stretch (arm up) 3. Side Bend (hands behind head) 4. Trunk Rotation, Arms Crossed 5. Trunk Rotation, Arms Extended | 1. Side Bend to Floor 2. Supported Reach Forward 3. Forward Bend to Floor 4. Towel Calf Stretch |
| **Seated balance** | | |
| 1. Single Arm Movement 2. Bilateral Arm Movement 3. Trunk Rotation, Hands Clasped 4. Supported Ant/Post Wt Shift: Lower Trunk Leading 5. Forward Lean for Sit to Stand 6. Unsupported Ant/Post Wt Shift: Upper Trunk Leading | 1. Unsupported Ant/Post Wt shift: Lower Trunk Leading 2. Supported Lateral Wt Shift: Upper Trunk Leading 3. Supported Lateral Wt Shift: Lower Trunk Leading 4. Unsupported Lateral Wt Shift: Lower Trunk Leading 5. Supported Diagonal Wt Shift | 1. Unsupported Diagonal Wt Shift 2. Reaching/Placing Objects in Diagonal Pattern 3. Side Scooting 4. Supported Seated Walk 5. Unsupported Seated Walk 6. Throwing/Catching Balloon |

^*^ = exercises with which a weight can be prescribed

**Additional File 1: List of all exercises that could be selected for Functional Maintenance Program**

| **Standing strengthening** | | |
| --- | --- | --- |
| 1. Shoulder Flexion^*^ 2. Shoulder Abduction^*^ 3. Arm Swing^*^ 4. Diagonal Arm Pull Down with Resistive Band 5. Diagonal Arm Pull Up with Resistive Band | 1. Wall Push-Ups 2. Standing Bilateral Heel Raise 3. Standing Toe Raise 4. Alternating Heel Raise and Toe Raise 5. Partial Knee Bend 6. Knee Bend | 1. Wall Squats 2. Forward Lunge 3. Sideways Lunge 4. Step-ups 5. Hip Abduction^*^ 6. Hip Extension^*^ |
| **Standing flexibility** | | |
| 1. Scapular Retraction 2. Lumbar Extension in Standing | 1. Trunk Twist 2. Supported Soleus Stretch | 1. Supported Gastroc Stretch |
| **Standing balance** | | |
| 1. Leg Swing 2. Supported Stepping on Spot 3. Supported High Stepping 4. Supported Grapevine 5. Supported Calf Raise 6. Ankle Strategies 7. Hip Strategies 8. Skiing on Spot 9. Diagonal Weight Shift | 1. Arm Motion with Feet Apart/Together/Partial Heel Toe or Heel Toe 2. Head Turn and Reach in Partial Heel Toe 3. Head Turn and Reach in Heel Toe 4. Varied Arm Positions in Heel Toe | 1. Single Step: Fwd/Back 2. Single Step: Side 3. Turning on Spot 4. Placing foot on Step in Front or to Side 5. Moving Object From floor To Table in Diagonal 6. Throwing/Catching Balloon 7. Ball Kick: Single Leg |
| **Gait balance/endurance** | | |
| 1. Walking 2. Stop/Start on Commnad 3. Walking with Up/Down Head Motion 4. Walking with Side to Side Head Motion 5. Walking Forward with 180° Turns | 1. Walking forward with 360° Turns 2. Walking Figure Eight 3. Picking Up/Carrying Object 4. Backward Walking 5. Fwd/Backward Progression With 180° Turns | 1. Side-Stepping 2. Crossovers 3. Grapevine 4. Obstacle Course 5. Stairs |

^*^ = exercises with which a weight can be prescribed

**Additional File 1: List of all exercises that could be selected for Functional Maintenance Program**
